# Pharmacist interventions for appropriate COVID-19 antiviral therapy in long-term care facilities: A public health initiative

**DOI:** 10.1017/ash.2023.246

**Published:** 2023-09-29

**Authors:** Jenna Preusker, Daniel Schroeder, Scott Bergman, Mark Rupp, Brandon Scott, Trevor Van Schooneveld, Andrew Watkins, Matthew Donahue, Muhammad Salman Ashraf

## Abstract

**Background:** Prescribing errors related to the COVID-19 oral antiviral agent nirmatrelvir-ritonavir have been reported and are primarily due to improper renal dosing and significant drug–drug interactions. These patient safety issues are particularly concerning in the long-term care facility (LTCF) population. The Nebraska Antimicrobial Stewardship Assessment and Promotion Program (ASAP) is a unique collaborative partnership involving the University of Nebraska Medical Center, Nebraska Medicine, and the Nebraska Department of Health and Human Services (DHHS). ASAP is funded through the Nebraska DHHS healthcare-associated infections and antimicrobial resistance (HAI/AR) program and was established in 2016, with a primary focus of promoting safe and effective antimicrobial use in Nebraska. In 2022, ASAP developed a statewide pharmacist-led service to assist LTCFs in evaluating prescriptions for COVID-19 oral therapeutics. We studied the impact of ASAP pharmacist intervention on COVID-19 oral antiviral prescriptions. **Methods:** ASAP created a centralized LTCF treatment request process for oral antivirals. A REDCap survey hosted on a dedicated program webpage was used to collect requests for treatment submitted by any LTCF in Nebraska, including assisted living facilities. An ASAP pharmacist reviewed each survey submission for renal and hepatic function, drug–drug interactions, date of symptom onset, and ability to take oral medications. After pharmacist approval, delivery of the appropriate COVID-19 therapeutic to the LTCF was coordinated with the dispensing pharmacy. The pharmacists recorded the specific interventions for each treatment in the program database. Descriptive analyses were used to study the program impact. **Results:** In total, 630 courses of oral COVID-19 antivirals were administered to Nebraska LTCF residents through the ASAP program in 2022. The median patient age was 84 years, and 59% were female. Most dispensed courses (n = 410, 65%) needed pharmaceutical interventions upon review for 506 individual interventions. The most frequent intervention was to hold or adjust doses of concomitant medications in 205 patients (33%), followed by antiviral dose adjustment for renal function in 117 patients (19%), and selecting an alternative COVID-19 therapy due to drug–drug interactions in 108 patients (17%). COVID-19 therapeutic agents were changed upon ASAP intervention to be in compliance with the National Institute of Health COVID-19 treatment guidelines in 37 patients (6%). **Conclusions:** Pharmacist review of oral antiviral prescriptions for COVID-19 through a public health–supported initiative identified and prevented potential patient safety issues in LTCF residents. Future studies should analyze the impact of similar interventions on patient outcomes.

**Figure:**
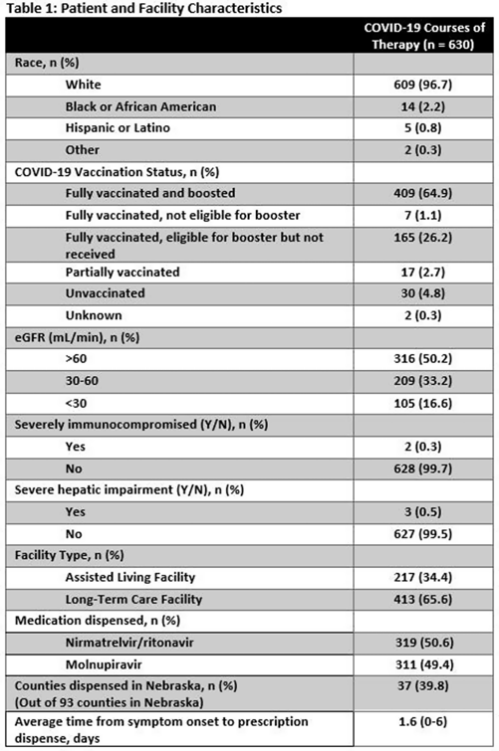

**Disclosures:** None

