# Pharmacist interventions for appropriate COVID-19 antiviral therapy in long-term care facilities: A public health initiative – ERRATUM

**DOI:** 10.1017/ash.2024.22

**Published:** 2024-02-08

**Authors:** Jenna Preusker, Daniel Schroeder, Mounica Soma, Scott Bergman, Mark Rupp, Brandon Scott, Trevor Van Schooneveld, Andrew Watkins, Matthew Donahue, Muhammad Salman Ashraf

In the above article^1^, Mounica Soma was omitted from the list of authors. The correct list of authors is Jenna Preusker, Daniel Schroeder, Mounica Soma, Scott Bergman, Mark Rupp, Brandon Scott, Trevor Van Schooneveld, Andrew Watkins, Matthew Donahue and Muhammad Salman Ashraf. The original article has been updated.

We apologize for the error.

## References

[1] Preusker J , Schroeder D , Soma M , et al. Pharmacist interventions for appropriate COVID-19 antiviral therapy in long-term care facilities: A public health initiative. Antimicrob Steward Healthc Epidemiol 2023;3(S2):s24–s25. 10.1017/ash.2024.22 PMC10897711 38415087

